# Diagnostic utility of metabolic parameters on FDG PET/CT for lymph node metastasis in patients with cN2 non-small cell lung cancer

**DOI:** 10.1186/s12885-021-08688-6

**Published:** 2021-09-02

**Authors:** Keita Nakanishi, Shota Nakamura, Tomoshi Sugiyama, Yuka Kadomatsu, Harushi Ueno, Masaki Goto, Naoki Ozeki, Takayuki Fukui, Shingo Iwano, Toyofumi Fengshi Chen-Yoshikawa

**Affiliations:** 1 Department of Thoracic Surgery, Nagoya University Graduate School of Medicine, 65 Tsurumai-cho, Showa-ku, Nagoya, 466-8550 Japan; 2 Department of Radiology, Nagoya University Graduate School of Medicine, Nagoya, Japan

**Keywords:** Non-small cell lung cancer, FDG-PET/CT, Lymph node metastasis, Metabolic parameter

## Abstract

**Background:**

The aim of this study was to assess the diagnostic utility of metabolic parameters on fluorine-18-fluoro-2-deoxy-D-glucose-positron emission tomography (FDG-PET)/computed tomography (CT) for predicting lymph node (LN) metastasis in patients with cN2 non-small cell lung cancer (NSCLC).

**Methods:**

We retrospectively reviewed patients who underwent surgery for cN2 NSCLC between 2007 and 2020. Those who had clinically diagnosed positive hilar and mediastinal LNs by routine CT and PET/CT imaging were investigated. To measure the metabolic parameters of LNs, the data according to maximum standardized uptake value (SUVmax), metabolic tumor volume (MTV), total lesion glycolysis (TLG), and LN-to-primary tumor ratio of SUVmax (LPR) were examined. The diagnosis of each retrieved LN was confirmed based on histopathological examination of surgical tissue specimens. Receiver operating characteristics (ROC) curves with area under the curve (AUC) calculations and multivariate analysis by logistic regression were performed.

**Results:**

Forty-five patients with 84 clinically diagnosed positive hilar or mediastinal LNs were enrolled in the present study. Of the 84 LNs, 63 LNs were pathologically proven as positive (75%). The SUVmax, MTV, TLG, and LPR of LN metastasis were significantly higher than those of benign nodes. In the ROC analysis, the AUC value of LPR [AUC, 0.776; 95% confidence interval (CI), 0.640–0.913] was higher than that of LN SUVmax (AUC, 0.753; 95% CI, 0.626–0.880) or LN TLG3.5 (AUC, 0.746; 95% CI, 0.607–0.885). Using the optimal LPR cutoff value of 0.47, the sensitivity, specificity, positive predictive value, negative predictive value, and accuracy were 84.1, 66.7, 88.3, 58.3, and 79.8%, respectively. Multivariate analysis by logistic regression showed that LPR was an independent predictor for LN metastasis (odds ratio, 6.45; 95% CI, 1.785–23.301; *P* = 0.004). In the subgroup analysis of adenocarcinoma patients (*n* = 18; 32 LNs), TLG3.5 was a better predictor (AUC, 0.816; 95% CI, 0.639–0.985) than LPR (AUC, 0.792; 95% CI, 0.599–0.986) or LN SUVmax (AUC, 0.792; 95% CI, 0.625–0.959).

**Conclusions:**

Our findings suggest that LPR on FDG-PET is a useful predictor for LN metastasis in patients with cN2 NSCLC. TLG can be a good predictor for LN metastasis in patients with adenocarcinoma.

**Supplementary Information:**

The online version contains supplementary material available at 10.1186/s12885-021-08688-6.

## Background

Accurate lymph node (LN) staging of primary non-small cell lung cancer (NSCLC), especially in patients with clinically suspected N2, is crucial in making therapeutic strategy decisions and in determining clinical outcomes [1–3]. In recent years, after receiving the results of the Pacific trial [4], the treatment strategy for stage III unresectable locally advanced NSCLC has changed dramatically, and the importance of assessing mediastinal LN metastasis accurately has increased. In clinical practice, computed tomography (CT) and/or fluorine-18-fluoro-2-deoxy-D-glucose-positron emission tomography (FDG-PET/CT) are usually performed for clinical LN staging. However, these modalities do not play a complete role in LN staging and causes diagnostic ambiguity (providing false-positive or false-negative results) in the clinical practice. Although LNs with a short-axis diameter of more than 10 mm on a contrast-enhanced CT are generally diagnosed as malignant, a previous meta-analysis showed a relatively low sensitivity (59%) and specificity (78%) [5]. In previous studies, using FDG-PET/CT in diagnosing mediastinal LNs also showed a relatively low sensitivity of 50–79% and specificity of 72–94% [6–10]. Inflammatory changes in LN due to tuberculosis or granulomatous disease can increase FDG uptake and false-positive results [6, 11]. On the contrary, a maximum standardized uptake value (SUVmax) threshold is associated with a greater number of false-negative findings [12, 13]. Mediastinal LN staging by endobronchial ultrasound guided-transbronchial needle aspiration (EBUS-TBNA)/mediastinoscopy was reported to be useful and was recommended when LN metastasis is suspected on imaging [14]. However, there are LNs, including those in stations #5 or #6, which are difficult to evaluate with EBUS-TBNA. Furthermore, there are possible complications, such as bleeding and mediastinitis [15]. Hence, a more accurate and less invasive diagnosis of LN involvement is required in patients with cN2 NSCLC.

In recent years, the usefulness of metabolic parameters, including metabolic tumor volume (MTV) and total lesion glycolysis (TLG) has been investigated. Several studies reported that MTV and TLG in the primary tumor of lung cancer, which provides information on tumor activity and volume, could predict the pathological tumor invasive size [16], LN status [17, 18], and prognosis [19–22] more accurately than SUVmax. Furthermore, the LN-to-primary tumor ratio of SUVmax (LPR) was also assessed to determine whether this parameter can be a good predictor for LN metastasis in NSCLC patients [13, 23]. However, only a few studies examined the diagnostic utility of these metabolic parameters and focused not on the primary tumor but on LNs for LN staging of primary lung cancer. Therefore, we aimed to investigate the role of metabolic parameters on FDG-PET/CT for LN metastasis in patients with cN2 NSCLC.

## Methods

### Patient selection

**Fig. 1:**
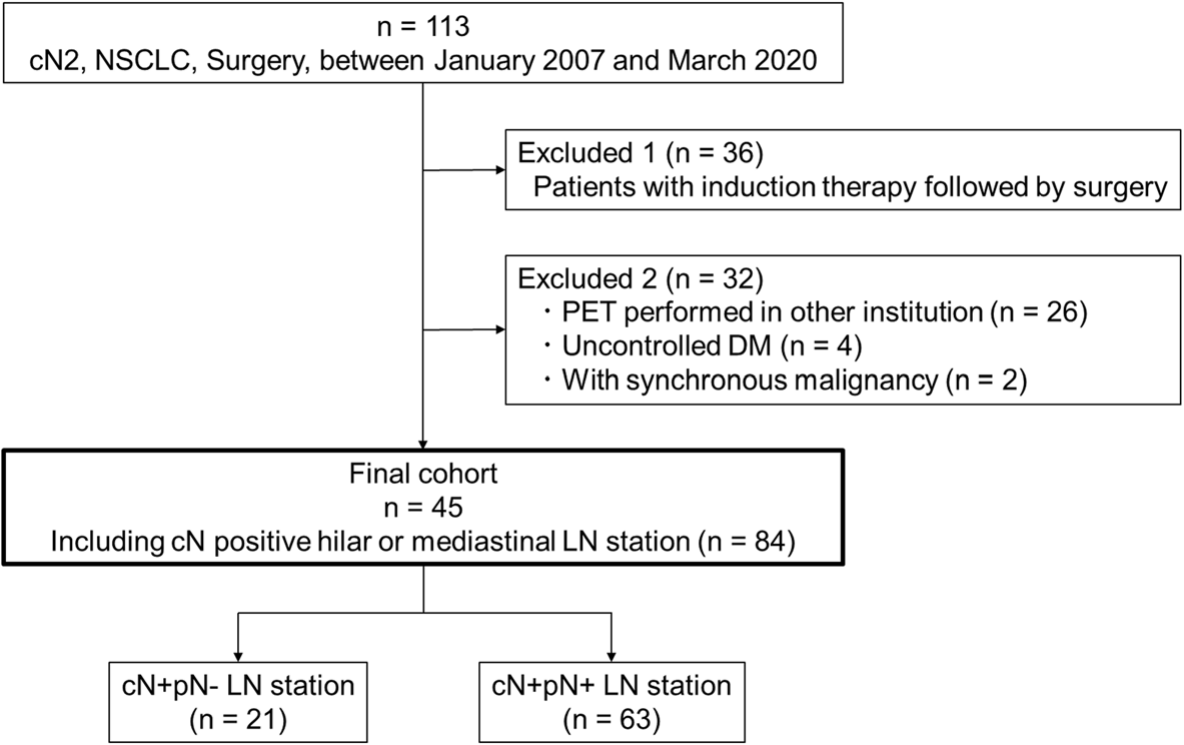
Patient selection. NSCLC, non-small cell lung cancer; PET, positron emission tomography; DM, diabetes mellitus; LN, lymph node

Overall, 2407 patients underwent surgery for primary NSCLC at Nagoya University Hospital between January 2007 and March 2020. Among them, 113 patients who underwent PET before surgery and upfront lung resection for cN2 NSCLC were retrospectively identified. As illustrated in Fig. 1, the following patients were excluded: those who received induction therapy (*n* = 36), who underwent PET in other institution (*n* = 26), with uncontrolled diabetes mellitus (*n* = 4), and with synchronous malignancy (*n* = 2). The present study was approved by the institutional review board of our institute (approval No. 2020–0375), and informed consent was obtained from each patient for the use of clinical data in various investigations.

### Data collection

Institutional databases and medical records of each patient were retrospectively reviewed to determine age, sex, tumor location, tumor size, clinical tumor-node-metastasis staging, histology, and surgical procedure. Staging was determined based on the seventh edition of the tumor, node, and metastasis classification for lung cancer [24]. LN stations were determined based on the International Association for the Study of Lung Cancer LN map [25]. At our institution, prior to treatment, LNs that were clinically diagnosed as positive were defined as measuring > 1.0 cm in the short-axis diameter on the CT image [5] with a SUVmax value of > 2.5 on PET/CT [26]. Although EBUS-TBNA was not mandatory in the present study, preoperative confirmation of N2 by EBUS-TBNA was performed in 10 patients (22%). The diagnosis of the retrieved LN was confirmed based on histopathological examination of surgical tissue specimens.

### FDG-PET/CT scan protocol and image evaluation

**Fig. 2:**
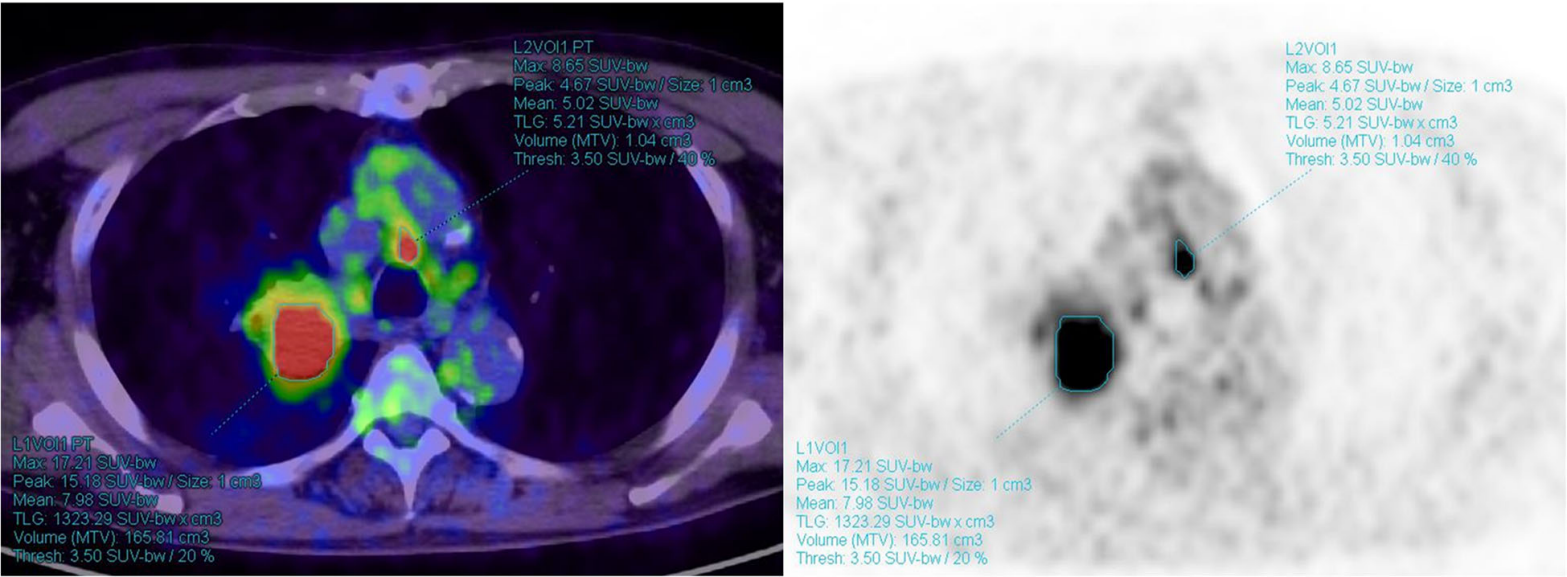
Representative example of the measurement of metabolic parameters on FDG-PET/CT for primary tumor and LNs. Primary tumor with an SUVmax of 17.21, MTV3.5 of 165.81 cm^3^, and TLG3.5 of 1323.29; #4R LN with an SUVmax of 8.65, MTV3.5 of 1.04 cm^3^, TLG3.5 of 5.21, and LPR of 0.5. In the pathological report, the LN was diagnosed as positive. FDG-PET/CT, fluorine-18-fluoro-2-deoxy-D-glucose-positron emission tomography; LN, lymph node; SUVmax, maximum standardized uptake value; MTV, metabolic tumor volume; TLG, total lesion glycolysis; LPR, lymph node-to-primary tumor ratio of SUVmax

FDG-PET/CT was performed as previously described [16]. PET/CT was carried out using a Biograph 16 scanner (Siemens Healthcare) within 31 days prior to surgery. The blood glucose levels were measured immediately before FDG injection. Patients with a blood glucose level greater than 150 mg/dL were not allowed to undergo PET/CT. The FDG dose was determined based on the body weight, using either 3.7 (for patients weighing < 60 kg) or 4.07 (for those weighing ≥60 kg) MBq/kg. Breath-holding and respiratory gating techniques were not applied. To measure the metabolic parameters, preexisting PET data were reanalyzed using the MM oncology software on a syngo.via workstation (Siemens Healthcare). All PET images were retrospectively evaluated by three investigators (K.N., S.N., and S.I.), including two certified thoracic surgeons and one radiologist specializing in chest radiology and nuclear medicine. The radiologist set the three-dimensional volume of interest to sufficiently cover the primary tumor and LNs clinically diagnosed as positive on a PET/CT images, and the data according to SUVmax, MTV, and TLG were automatically extracted (Fig. 2). The SUVmax was defined as the maximum value of the volume of interest. The LPR was calculated as LN SUVmax divided by primary tumor SUVmax. The MTV was defined as the tumor volume over a threshold value of SUV, which varied from 2.5 to 5.0. The TLG was obtained by multiplying MTV with its SUVmean, which varied from 2.5 to 5.0. All MTV and TLG threshold values from 2.5 to 5.0 in 0.5 increments were investigated during the study period. However, MTV3.5 and TLG3.5 were adopted in the analysis phase since these values were the most sensitive threshold values.

### Statistical analysis

The differences between groups were compared using the Mann-Whitney U test for continuous variables. Receiver operating characteristics (ROC) curves with area under the curve (AUC) calculations were performed to determine whether SUVmax, MTV, TLG, and LPR can predict nodal status and to determine the optimal cutoff values by Youden’s Index. A multivariate logistic regression analysis that considered significant risk factors from ROC analysis, except for the factor with multicollinearity, was performed. For all analyses, *P*-values of < 0.05 were considered statistically significant. All statistical analyses were performed using the SPSS Statistics 25 software (IBM Corporation, Armonk, NY).

## Results

Forty-five patients with 84 clinically diagnosed positive hilar or mediastinal LNs were enrolled in the present study. The clinicopathological characteristics are summarized in Table 1. All cohorts comprised 39 men (87%) and six women, with a median age of 70 years (40–79). The median tumor size was 35 mm (9–106 mm), and the clinical T factor was mainly T1–T2 (73%). The primary histology was adenocarcinoma in 18 patients (40%), squamous cell carcinoma in 18 patients (40%), and others including adenosquamous cell carcinoma or large cell carcinoma and neuroendocrine tumors in nine patients (20%). In the present study, lobectomy was mainly performed (76%), and systemic LN dissection of more than ND2a-2 was performed on 36 patients (80%).

**Table 1:** Baseline characteristics

Characteristics	*n* = 45 (%)
Age, median (range)	70 (40–79)
Sex, male	39 (87)
Tumor location	
RUL	16 (36)
RML	1 (2)
RLL	12 (27)
LUL	11 (24)
LLL	3 (7)
RMLL	2 (4)
Tumor size, mm, median (range)	35 (9–106)
Clinical T factor	
T1	16 (35)
T2	17 (38)
T3	8 (18)
T4	4 (9)
Histology	
Adenocarcinoma	18 (40)
Squamous cell carcinoma	18 (40)
Others ^a^	9 (20)
Procedure of operation	
Lobectomy	34 (76)
Bi-lobectomy	7 (16)
Pneumonectomy	2 (4)
No resection (only LN sampling)	2 (4)
Lymph node dissection	
Only LN sampling	2 (4)
ND2a-1	7 (16)
ND2a-2	34 (76)
ND2b	2 (4)

*RUL* right upper lobe, *RML* right middle lobe, *RLL* right lower lobe, *LUL* left upper lobe, *LLL* left lower lobe, *RMLL* right middle and lower lobe, *LN* lymph node, *ND* node dissection

^a^ Including adenosquamous cell carcinoma, large cell carcinoma and neuroendocrine tumors

**Fig. 3:**
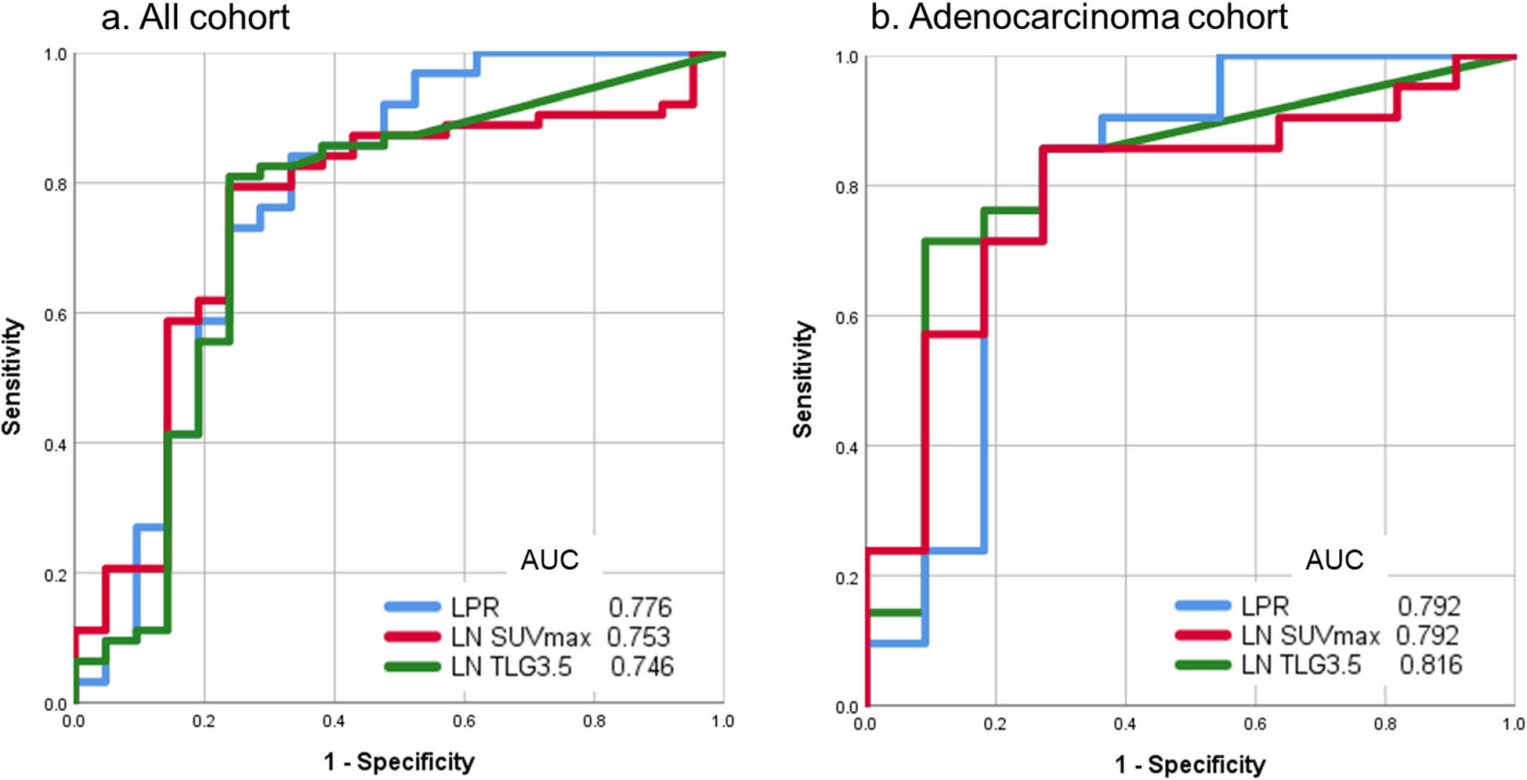
Receiver operating characteristic analysis of all cohorts (**a**) and of adenocarcinoma cohort (**b**). AUC, area under the curve; LPR, lymph node-to-primary tumor ratio of SUVmax; LN, lymph node; SUVmax, maximum standardized uptake value; TLG, total lesion glycolysis

Of the 84 hilar or mediastinal LNs clinically diagnosed as positive, 63 were pathologically proven as positive (75%), whereas 21 were pathologically proven as negative (25%). The FDG-PET/CT parameters for negative and positive LNs are presented in Table 2. The SUVmax, MTV, TLG, and LPR of metastatic LNs were higher than those of benign nodes. No significant difference was observed in the primary tumor SUVmax. In the ROC analysis, the AUC value of LPR [AUC, 0.776; 95% confidence interval (CI), 0.640–0.913] was higher than that of LN SUVmax (AUC, 0.753; 95% CI, 0.626–0.880) or LN TLG3.5 (AUC, 0.746; 95% CI, 0.607–0.885) (Fig. 3a). MTV had the lowest AUC value compared with the above parameters (not shown in Fig. 3a). Using the optimal LPR cutoff value of 0.47, the sensitivity, specificity, positive predictive value, negative predictive value, and accuracy were 84.1, 66.7, 88.3, 58.3, and 79.8%, respectively. On the contrary, the LN SUVmax (cutoff value, 4.15) and LN TLG3.5 (cutoff value, 1.26) showed sensitivity values of 79.4 and 81.0%, specificity values of 76.2 and 76.2%, positive predictive values of 90.9 and 91.1%, negative predictive values of 55.2 and 57.1%, and accuracies of 78.6 and 79.8%, respectively. The results of the multivariate analysis of predictors associated with LN metastasis by logistic regression are shown in Table 3. LPR was the independent predictor for LN metastasis (odds ratio, 6.45; 95% CI, 1.785–23.301; *P* = 0.004).

**Table 2:** FDG-PET/CT parameters for negative and positive LNs

Characteristics	Negative LNs (*n* = 21)	Positive LNs (*n* = 63)	*P* value
LN location			
N2 nodes			
Superior mediastinal nodes			
#2R	2	0	
#3p	1	1	
#4R	6	14	
#4 L	0	3	
Aortic nodes			
#5-#6	0	10	
Inferior mediastinal nodes			
#7	4	15	
#8-#9	0	2	
N1 nodes			
#10	2	3	
#11	4	9	
#12	2	6	
Primary tumor SUVmax	10.66 (2.55–17.91)	9.04 (1.66–18.67)	0.09
LN SUVmax	3.54 (2.52–11.20)	5.77 (2.81–15.54)	0.001
LN MTV3.5	0.01 (0–13.36)	1.44 (0–22.68)	0.001
LN TLG3.5	0.05 (0–71.25)	6.34 (0–162.78)	0.001
LPR	0.37 (0.15–1.76)	0.81 (0.26–2.26)	< 0.001

*FDG-PET/CT* fluorine-18-fluoro-2-deoxy-D-glucose positron emission tomography, *LN* lymph node, *SUVmax* maximum standardized uptake value, *MTV* metabolic tumor volume, *TLG* total lesion glycolysis, *LPR* lymph node-to-primary tumor ratio of SUVmax

**Table 3:** Multivariate analysis of predictors associated with LN metastasis by logistic regression

Characteristics	OR	95%CI	*P* value
LN SUVmax	1.037	0.053–20.207	0.98
LN TLG3.5	8.727	0.459–166.02	0.15
LPR	6.450	1.785–23.301	0.004

*LN* lymph node, *SUVmax* maximum standardized uptake value, *TLG* total lesion glycolysis, *LPR* lymph node-to-primary tumor ratio of SUVmax, *OR* odds ratio, *CI* confidence interval

A subgroup analysis based on the histology of primary tumor was performed. In the subgroup analysis of patients with adenocarcinoma (*n* = 18; 32 LNs), the SUVmax, MTV, TLG, and LPR of metastatic LNs were higher than those of benign nodes, but there was no significant difference in primary tumor SUVmax (Supplementary Table S1). In the subgroup analysis of patients with squamous cell carcinoma (*n* = 18; 34 LNs), the SUVmax and LPR of metastatic LNs were higher than those of benign nodes, but there were no significant differences in primary tumor SUVmax, MTV and TLG of LNs (Supplementary Table S2). In the ROC analysis based on adenocarcinoma, TLG3.5 was a better predictor (AUC, 0.816; 95% CI, 0.639–0.985) than LPR (AUC, 0.792; 95% CI, 0.599–0.986) or LN SUVmax (AUC, 0.792; 95% CI, 0.625–0.959) (Fig. 3b). Using the optimal TLG3.5 cutoff value of 1.26, the sensitivity, specificity, positive predictive value, negative predictive value, and accuracy were 71.4, 90.9, 93.8, 62.5, and 78.1%, respectively. In the ROC analysis based on squamous cell carcinoma, LPR was a better predictor (AUC, 0.831; 95% CI, 0.642–1.000) than LN SUVmax (AUC, 0.804; 95% CI, 0.593–1.000) or TLG3.5 (AUC, 0.701; 95% CI, 0.436–0.966). On the other hand, there was no predictor in other histological group. A subgroup analysis based on the LN location was performed. All LNs were assigned to the following two groups: N2 nodes (*n* = 58) and N1 nodes (*n* = 26); PET parameters were compared between these groups. The LN SUVmax, MTV, and TLG of N1 nodes were significantly higher than those of N2 nodes, but there was no significant difference in the LPR between the two groups (Supplementary Table S3). In the ROC analysis, the AUC value of LPR was higher than that of the LN SUVmax or TLG3.5 in the N2 nodes as well as in the entire cohort (AUC, 0.908; 95% CI, 0.821–0.995) (Supplementary Fig. S1). On the other hand, there was no significant predictor in the N1 nodes.

## Discussion

The present study suggested that LPR on FDG-PET before surgery, and not SUVmax or TLG, is the best predictor of LN metastasis in patients with cN2 NSCLC. TLG can be a good predictor for LN metastasis in patients with adenocarcinoma. To the best of our knowledge, this is the first study to investigate metabolic parameters such as MTV or TLG for the diagnosis of LN metastasis. This study evaluated the presence of LN metastasis through surgical pathology, including #5 or #6 LN station, which was difficult to evaluate with EBUS-TBNA.

In patients with NSCLC, accurate preoperative LN evaluation is required, especially in those with clinically suspected mediastinal LN metastasis. This is because the status of mediastinum LNs determines the patient’s treatment strategies or modalities. The present study demonstrated that the sensitivity and specificity for LN metastasis were 84.1 and 66.7% using LPR, which could reduce the false-negative findings due to its high sensitivity. In other words, LPR was useful for the exclusionary diagnosis of LN metastasis. These results might highlight the issue of false-positive test results, which resulted from a small volume of benign LN being present or relatively high metabolic activity or both. Several previous studies reported the usefulness of LPR when assessing LN status [13, 23]. In a series of 44 patients with 92 mediastinal LNs with a SUVmax of 2.5–4.0, Moloney et al. reported that the LPR of 0.3 was the optimal cutoff value for predicting malignancy (sensitivity, 91%; specificity, 71%) [13]. In a series of 172 patients with 504 PET-positive LNs, Mattes and colleagues found that a cutoff value of 0.28 was optimal (sensitivity, 93%; specificity, 87%) [23]. Their optimal cutoff values were somewhat lower than that of the present study, which was 0.47. This was most likely due to study population differences. For example, the former study excluded LNs with a SUVmax of more than 4.0, while the latter study included LNs with a SUV max of less than 2.5. Although there were differences in the inclusion criteria, background, and rate of LN malignancy, these results, according to LPR with a high sensitivity and a relatively low specificity, supported our results.

On the contrary, using LPR reduced the specificity and increased the number of false-positive cases. This result might be associated with the concurrent infectious disease such as obstructive pneumonia and granulomatous inflammation owing to the relatively high prevalence of tuberculosis in our ethnic background. According to predictors for LN metastasis using the SUV threshold, previous studies showed high specificity but relatively low sensitivity [6–10]. Darling and colleagues reported the accuracy of PET-CT in 149 patients who underwent mediastinoscopy and/or thoracotomy for mediastinal staging [9]. Although their study had PET-CT sensitivity of 70% and specificity of 94%, they highlighted the risk of missing the chance of surgery because of the relatively high number of false-positive findings. There are limitations in comparing the sensitivities, specificities, and other parameters between studies due to the heterogeneity of the prevalence of malignancies, ethnic background, tumor histology, and cutoff values. However, including the LPR into the diagnostic approach could increase the accuracy for predicting LN metastasis. Indeed, in a subgroup analysis, the AUC value of the LPR was higher than that of the LN SUVmax or TLG3.5, especially in N2 nodes. We considered that tumor volume had an effect on hilar LNs, but SUV uptake rather than tumor volume may have had a stronger effect on mediastinal LNs. Therefore, LPR was the best predictor of LN metastasis, especially in N2 nodes.

In recent years, the usefulness of metabolic parameters, including MTV and TLG, has been investigated. Several studies could predict the LN status according to MTV and TLG in the primary tumor of lung cancer [17, 18]. However, no studies have investigated metabolic parameters of LN itself, and whether these parameters were useful remains unclear. We also investigated the usefulness of metabolic parameters, and revealed the association between those parameters and pathological invasive size in patients with adenocarcinoma [16]. Initially, we assumed that these metabolic parameters would have a more diagnostic value in predicting LN, as well as the primary tumor, but the results were contrary to that hypothesis. This might be because, unlike the primary tumor, the metabolic activity in the LN was lower and the difference in LN volume was less apparent. In cases of adenocarcinoma, TLG had a higher diagnostic value than LPR. Although the mechanism was unknown, tumor activity or glucose transporter family expression might be different at the metastatic site depending on the tissue type [27]. Hence, further studies are warranted to clarify this mechanism.

The present study has several limitations. First, our retrospective study revealed a single-center investigation conducted in individuals with the same ethnic background and geographical region. Therefore, the insufficiency of data and external validity are potential problems. Second, there was one limitation to confirm that the LN evaluated on imaging was the same LN diagnosed pathologically in any study of this nature. Third, our study had a small sample size. PET scans performed at other institutions were excluded to ensure uniform FDG-PET/CT imaging accuracy. A further prospective multicenter study is needed to assess not only the diagnostic ability but also the effect of metabolic parameters on making treatment decisions.

## Conclusions

Our findings suggest that LPR on FDG-PET is a useful predictor for LN metastasis in patients with cN2 NSCLC. In addition, TLG can be a good predictor for LN metastasis in patients with adenocarcinoma. Further prospective, multi-institutional study is needed to validate the outcomes of the present study and to assess the effect of metabolic parameters on the therapeutic strategy.

## Supplementary Information

**Additional file 1 **: **Supplementary Table S1**. FDG-PET/CT parameters for negative and positive LNs in adenocarcinoma. FDG-PET/CT, fluorine-18-fluoro-2-deoxy-D-glucose positron emission tomography/ computed tomography; LN, lymph node; SUVmax, maximum standardized uptake value; MTV, metabolic tumor volume; TLG, total lesion glycolysis; LPR, lymph node-to-primary tumor ratio of SUVmax. **Supplementary Table S2.** FDG-PET/CT parameters for negative and positive LNs in squamous cell carcinoma. FDG-PET/CT, fluorine-18-fluoro-2-deoxy-D-glucose positron emission tomography/ computed tomography; LN, lymph node; SUVmax, maximum standardized uptake value; MTV, metabolic tumor volume; TLG, total lesion glycolysis; LPR, lymph node-to-primary tumor ratio of SUVmax. **Supplementary Table S3**. FDG-PET/CT parameters according to LN location. FDG-PET/CT, fluorine-18-fluoro-2-deoxy-D-glucose positron emission tomography/ computed tomography; LN, lymph node; SUVmax, maximum standardized uptake value; MTV, metabolic tumor volume; TLG, total lesion glycolysis; LPR, lymph node-to-primary tumor ratio of SUVmax.

**Additional file 2 **: **Supplementary Figure S1.** Receiver operating characteristic analysis of N2 nodes (Figure S1a) and of N1 nodes (Figure S1b). AUC, area under the curve; LPR, lymph node-to-primary tumor ratio of SUVmax; LN, lymph node; SUVmax, maximum standardized uptake value; TLG, total lesion glycolysis.

## Data Availability

The datasets used and/or analysed during the current study available from the corresponding author on reasonable request.

## Abbreviations

LN: Lymph node
NSCLC: Non-small cell lung cancer
CT: Computed tomography
FDG-PET: Fluorine-18-fluoro-2-deoxy-D-glucose-positron emission tomography
SUV: Standardized uptake value
EBUS-TBNA: Endobronchial ultrasound guided-transbronchial needle aspiration
MTV: Metabolic tumor volume
TLG: Tumor lesion glycolysis
LPR: Lymph-to-primary tumor ratio of SUVmax
ROC: Receiver operating characteristics
AUC: Area under the curve
