# Supplementary material for: Diagnostic utility of metabolic parameters on FDG PET/CT for lymph node metastasis in patients with cN2 non-small cell lung cancer
Source: BMC Cancer. 2021 Sep 2;21:983. doi: 10.1186/s12885-021-08688-6 (PMC8414769; doi:10.1186/s12885-021-08688-6)
Supplement: Supplementary file 1 — Additional file 1 : Supplementary Table S1. FDG-PET/CT parameters for negative and positive LNs in adenocarcinoma. FDG-PET/CT, fluorine-18-fluoro-2-deoxy-D-glucose positron emission tomography/ computed tomography; LN, lymph node; SUVmax, maximum standardized uptake value; MTV, metabolic tumor volume; TLG, total lesion glycolysis; LPR, lymph node-to-primary tumor ratio of SUVmax. Supplementary Table S2. FDG-PET/CT parameters for negative and positive LNs in squamous cell carcinoma. FDG-PET/CT, fluorine-18-fluoro-2-deoxy-D-glucose positron emission tomography/ computed tomography; LN, lymph node; SUVmax, maximum standardized uptake value; MTV, metabolic tumor volume; TLG, total lesion glycolysis; LPR, lymph node-to-primary tumor ratio of SUVmax. Supplementary Table S3. FDG-PET/CT parameters according to LN location. FDG-PET/CT, fluorine-18-fluoro-2-deoxy-D-glucose positron emission tomography/ computed tomography; LN, lymph node; SUVmax, maximum standardized uptake value; MTV, metabolic tumor volume; TLG, total lesion glycolysis; LPR, lymph node-to-primary tumor ratio of SUVmax. [file 12885_2021_8688_MOESM1_ESM.docx]

Diagnostic utility of metabolic parameters on FDG PET/CT for lymph node metastasis in patients with cN2 non-small cell lung cancer

Authors: Keita Nakanishi, MD, ^1^ Shota Nakamura, MD, ^1^ Tomoshi Sugiyama, MD, ^1^ Yuka Kadomatsu, MD, ^1^ Harushi Ueno, MD, ^1^ Masaki Goto, MD, ^1^ Naoki Ozeki, MD, ^1^ Takayuki Fukui, MD, ^1^ Shingo Iwano, MD, ^2^ and Toyofumi Fengshi Chen-Yoshikawa, MD ^1^

Affiliations and addresses of the authors:

1) Department of Thoracic Surgery, Nagoya University Graduate School of Medicine, Nagoya, Japan

2) Department of Radiology, Nagoya University Graduate School of Medicine, Nagoya, Japan

Address correspondence to:

Shota Nakamura, MD, Department of Thoracic Surgery, University Graduate School of Medicine, 65 Tsurumai-cho, Showa-ku, Nagoya, 466-8550, Japan

Tel: +81-52-744-2375; Fax: +81-52-744-2382

E-mail: shota197065@med.nagoya-u.ac.jp

Supplementary Table S1. FDG-PET/CT parameters for negative and positive LNs in adenocarcinoma

| Characteristics | Negative LNs  ( n = 11) | Positive LNs  ( n = 21) | *P* value |
| --- | --- | --- | --- |
| Primary tumor SUVmax | 8.90 (2.55-17.91) | 7.59 (1.66-16.63) | 0.19 |
| LN SUVmax | 3.39 (2.52-9.36) | 5.97 (2.88-14.82) | 0.006 |
| LN MTV3.5 | 0 (0-9.01) | 0.8 (0-17.63) | 0.003 |
| LN TLG3.5 | 0 (0-49.75) | 3.77 (0-125.50) | 0.003 |
| LPR | 0.44 (0.15-1.76) | 0.90 (0.35-2.26) | 0.006 |

FDG-PET/CT, fluorine-18-fluoro-2-deoxy-D-glucose positron emission tomography/ computed tomography; LN, lymph node; SUVmax, maximum standardized uptake value; MTV, metabolic tumor volume; TLG, total lesion glycolysis; LPR, lymph node-to-primary tumor ratio of SUVmax

Supplementary Table S2. FDG-PET/CT parameters for negative and positive LNs in squamous cell carcinoma

| Characteristics | Negative LNs  ( n = 7) | Positive LNs  ( n = 26) | *P* value |
| --- | --- | --- | --- |
| Primary tumor SUVmax | 10.66 (9.04-17.91) | 9.59 (4.17-18.67) | 0.16 |
| LN SUVmax | 3.89 (3.25-9.42) | 5.92 (2.81-15.54) | 0.01 |
| LN MTV3.5 | 0.17 (0-13.36) | 1.83 (0-22.68) | 0.11 |
| LN TLG3.5 | 0.63 (0-71.25) | 8.12 (0-162.78) | 0.11 |
| LPR | 0.34 (0.19-0.88) | 0.70 (0.35-1.41) | 0.006 |

FDG-PET/CT, fluorine-18-fluoro-2-deoxy-D-glucose positron emission tomography/ computed tomography; LN, lymph node; SUVmax, maximum standardized uptake value; MTV, metabolic tumor volume; TLG, total lesion glycolysis; LPR, lymph node-to-primary tumor ratio of SUVmax

Supplementary Table S3. FDG-PET/CT parameters according to LN location

| Characteristics | N2 nodes  ( n = 58) | N1 nodes  ( n = 26) | *P* value |
| --- | --- | --- | --- |
| LN SUVmax | 4.91 (2.52-15.54) | 6.55 (2.81-15.14) | 0.04 |
| LN MTV3.5 | 0.61 (0-21.14) | 2.46 (0-22.68) | 0.02 |
| LN TLG3.5 | 2.43 (0-143.04) | 13.42 (0-162.78) | 0.02 |
| LPR | 0.62 (0.15-2.26) | 0.86 (0.19-1.92) | 0.10 |

FDG-PET/CT, fluorine-18-fluoro-2-deoxy-D-glucose positron emission tomography/ computed tomography; LN, lymph node; SUVmax, maximum standardized uptake value; MTV, metabolic tumor volume; TLG, total lesion glycolysis; LPR, lymph node-to-primary tumor ratio of SUVmax
