# Supplementary figures and images for: Diagnostic utility of metabolic parameters on FDG PET/CT for lymph node metastasis in patients with cN2 non-small cell lung cancer
Source: BMC Cancer. 2021 Sep 2;21:983. doi: 10.1186/s12885-021-08688-6 (PMC8414769; doi:10.1186/s12885-021-08688-6)

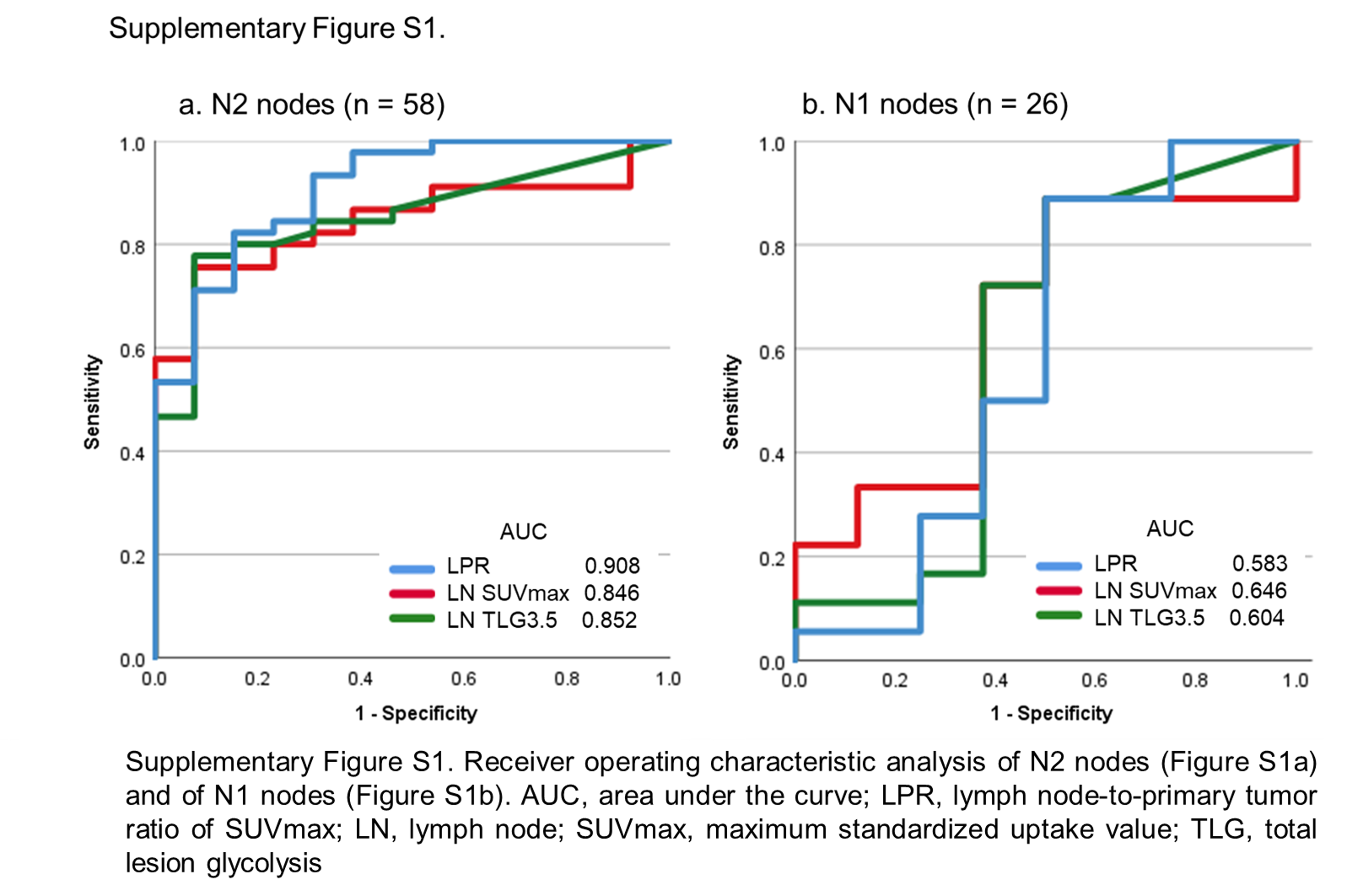

Supplement: Supplementary file 2 — Additional file 2 : Supplementary Figure S1. Receiver operating characteristic analysis of N2 nodes (Figure S1a) and of N1 nodes (Figure S1b). AUC, area under the curve; LPR, lymph node-to-primary tumor ratio of SUVmax; LN, lymph node; SUVmax, maximum standardized uptake value; TLG, total lesion glycolysis. [file 12885_2021_8688_MOESM2_ESM.tif]
